# Heat shock cognate protein 70 encodes antigenic epitopes recognised by HLA-B4601-restricted cytotoxic T lymphocytes from cancer patients

**DOI:** 10.1038/sj.bjc.6601203

**Published:** 2003-09-09

**Authors:** K Azuma, S Shichijo, H Takedatsu, N Komatsu, H Sawamizu, K Itoh

**Affiliations:** 1 Department of Immunology, Kurume University School of Medicine, 67 Asahi-machi, Kurume, Fukuoka 830-0011, Japan

**Keywords:** CTL, HLA-B46, tumour epitopes, heat shock cognate protein 70, cancer vaccine

## Abstract

Heat shock cognate protein 70 (HSC70), a highly conserved protein and a member of the family of molecular chaperones, has the ability to induce cytotoxic T lymphocyte (CTL) responses through binding and carrying antigenic peptides. We demonstrated in this study that the *HSC70* gene encodes two antigenic peptides recognised by HLA-B46-restricted and tumour-reactive CTLs established from tumour-infiltrating lymphocytes of a colon cancer. These HSC70-derived peptides, at amino-acid positions 106–114 and 233–241, had the ability to induce HLA-B46-restricted and peptide-specific CTLs, which are reactive to tumour cells, from peripheral blood mononuclear cells of the majority of epithelial cancer patients tested. These results, along with those from the previous studies, indicate the two ways of HSC70 involvement in the immune responses to tumours: chaperones and antigens, and thus may provide a new insight for the development of HSC70-directed cancer-specific immunotherapy.

Current modalities for cancer immunotherapy are rarely considered from the viewpoint of adaptive immunity. Therefore, the development of a new modality directed by adaptive immunity is needed. Many antigenic peptides recognised by HLA-A class I restricted cytotoxic T lymphocytes (CTLs) were identified during the past decade (van der Bruggen *et al*, 1991; Kawakami *et al*, 1994; Boon and van der Bruggen, 1996; Shichijo *et al*, 1998; Yang *et al*, 1999; Azuma *et al*, 2003), and several clinical trials using these antigenic peptides have been carried out (Wang *et al*, 1999; Jager *et al*, 2000; Gohara *et al*, 2002). However, tumour regression was rarely observed in such clinical studies, and the molecular basis of immunotherapy for cancer remains to be fully elucidated. One potential approach for better understanding of molecular basis of tumour immunology could be to identify antigenic peptides recognised by HLA-B class I-restricted CTLs. We recently established HLA-B4601-restricted and tumour-reactive CTL line from T lymphocytes infiltrating into colon tumours (Azuma *et al*, 2003). HLA-B4601 allele is exclusively expressed in Asians, and there is no such study except for our previous report. Subsequently, with that CTL line, we extended our study to identify antigenic epitopes capable of binding to HLA-B4601 allele; we report in this study that *heat shock cognate protein 70 (HSC70)* gene encodes two nonmutated nonapeptides with the ability to induce HLA-B4601-restricted and tumour-reactive CTLs from cancer patients. HSC70 was reported to elicit protective immunity to a diverse array of cancers mainly through binding and carrying of tumour-derived epitopes to CTLs (Blachere *et al*, 1997; Udono *et al*, 2001). We discussed in this study the possible contribution of the HSC70-derived peptides by themselves in induction of tumour-reactive CTLs.

## MATERIALS AND METHODS

### Tumour cell lines

The epithelial cancer cell lines used in this study were OSC20 (HLA-B46^+^, oral cancer), Ca9-22 (HLA-B46^+^, oral cancer), MKN45 (HLA-B46^+^, stomach cancer), Kuma-1 (HLA-B46^−^, head and neck cancer), SW620 (HLA-B46^−^, colon cancer), COLO320 (HLA-B46^−^, colon cancer), KWS (HLA-B46^−^, stomach cancer), COS-7 (SV40 transformed African Green monkey kidney cell), and Epstein–Barr virus (EBV)-transformed B cell line (EBV-B^3^) (HLA-B46^+^) established from peripheral blood mononuclear cells (PBMCs) of a colon cancer patient from whom the OKB-CTL line was established. Details of these cell lines were reported elsewhere (Azuma *et al*, 2003).

### Identification of the cDNA clone

The HLA-B46-restricted and tumour cell-reactive CTL (OKB-CTL) line was established from tumour-infiltrating lymphocytes (TILs) of a patient with colon cancer (HLA-A0207/3101, −B4601/5101, −Cw1) by incubation with interleukin-2 (IL-2) (100 U ml^−1^) alone for more than 50 days by the methods as reported previously (Ito *et al*, 2001). The surface phenotypes of the CTLs were investigated by immunofluorescence assay with anti-CD3, -CD4, -CD8, anti-CD16 (Nichirei, Tokyo, Japan), and anti-NkD2D (R&D Systems, Inc, Mckinley, NE, USA) monoclonal antibodies (mAbs) with FACScan as reported previously (Azuma *et al*, 2003).

The cDNA expression gene cloning method, described previously (Shichijo *et al*, 1998), was used to identify genes coding for tumour antigens recognised by the OKB-CTL. In brief, poly (A)^+^ RNA of the SW620 tumour cells was converted to cDNA, ligated to the *Sal*I adapter, and inserted into the expression vector pCMV-SPORT-2 (Invitrogen, San Diego, CA, USA). cDNAs of *HLA-B4601*, *-B5201*, *-B5101*, and -*A0207* were obtained by reverse transcription (RT)–polymerase chain reaction (PCR) and cloned into the eukaryotic expression vector pCR3 (Invitrogen). A measure of 200 ng of the plasmid DNA pools as well as the clones of the SW620 cDNA library were mixed with 200 ng of the *HLA-B4601* cDNA in 120 *μ*l of OPTI-MEM (Invitrogen) for 30 min. A 50 *μ*l aliquot of the mixture was then added to the COS-7 cells (5 × 10^3^), followed by incubation for 5 h. RPMI-1640 medium containing 10% foetal calf serum (FCS) was then added and cultured for 2 days followed by addition of the OKB-CTLs (5 × 10^4^ cells per well). After an 18-h incubation, 100 *μ*l of supernatant was collected and tested for the production of interferon-*γ* (IFN-*γ*) by enzyme-linked immuno-sorbent assay (ELISA) in a duplicate assay. DNA sequencing of a positive cDNA clone was performed with a dideoxynucleotide sequencing method using a DNA Sequencing kit (Perkin-Elmer, Foster, CA, USA) and analysed by an ABI PRISM™ 377 DNA Sequencer (Perkin-Elmer).

### Northern and Western blot analyses

Preparation of the RNA (5 *μ*g per lane), the transfer of RNA to nylon membranes, and the subsequent hybridisation processes have been described elsewhere (Shichijo *et al*, 1998). A ^32^P-labelled 704 bp fragment of *Acc*I-digested *HSC70* cDNA was used as a probe, which has 74% homology with the stress-inducible heat shock protein 70 (HSP70) at the nucleotide level. The membranes were washed four times and then autoradiographed. The expressions of HSC70 and HSP70 were investigated at the protein level by means of Western blotting with anti-Hsc70 (Product#:SPA-815, Stressgen, Victoria, Canada) and anti-Hsp70 (Product#:SPA-810, Stressgen) mAbs, respectively. HSP70 is a family of HSPs, and has 74 and 81% sequence homology with HSC70 at the nucleotide and amino-acid levels, respectively. The method for Western blotting was reported elsewhere (Yang *et al*, 1999).

### Peptides and CTL assay

Peptides capable of binding to the HLA-B4601 molecules were sought in the literature with regard to peptides for HLA-B4601-binding motifs (third amino acids (aa) were K, R, N, I, P, and F, and ninth aa were Y or F) (Barber *et al*, 1996), and the following 10 different peptides (>70% purity) were synthesised for screening (HSC70_33–41_, HSC70_59–67_, HSC70_69–77_, HSC70_106–114_, HSC70_136–144_, HSC70_233–241_, HSC70_237–245_, HSC70_346–354_, HSC70_451–459_, and HSC70_537–545_). For further studies, two peptides (HSC70_106–114_ and HSC70_233–241_ with >90% purity) were obtained. For the detection of antigenic peptides, COS-7 (5 × 10^3^) cells transfected with the *HLA-B4601*, *HLA-B5101*, or *HLA-B5201* cDNA were pulsed with each of 20 peptides at five different concentrations for 2 h, and then incubated with CTLs for 18 h. The supernatants were collected to measure IFN-*γ* by ELISA (limit of sensitivity: 10 pg ml^−1^) in a duplicate assay.

### Cytotoxic T lymphocyte induction from cancer patients

Peripheral blood mononuclear cells from 80 epithelial cancer patients (40 non-small-cell lung cancer patients, 30 prostate cancer patients, and 10 colon cancer patients) and also from 40 HDs were provided for screening of serological expression of HLA-B types. Subsequently, PBMCs from 10 HLA-B46^+^ cancer patients (five lung cancer and five prostate cancer patients) and four HLA-B46^+^ HD served as subjects for the CTL induction assay. Informed consent was obtained from all subjects. The HLA-class I of PBMCs was serotyped by conventional serological methods, as reported previously (Yang *et al*, 1999). The HLA-B alleles were also genotyped by the methods as reported previously (Azuma *et al*, 2003), and all of them were B4601, which was expected since the genotype of B46 in Asians is predominantly (>95%) a B4601 (Imanishi *et al*, 1992; Barber *et al*, 1996; Akesaka *et al*, 2000). For the induction of peptide-specific CTLs, PBMCs (1 × 10^5^ cells per well) were incubated with 10 *μ*M of each peptide in a round-bottom 96-well microculture plate (Nunc, Roskilde, Denmark) in 200 *μ*l culture medium containing IL-2, as reported previously (Maeda *et al*, 2002). On the fourth and seventh days of culture, half of the medium was replaced with a new medium containing a corresponding peptide. On the 10th day, the cells were harvested, washed, and tested in duplicate assays for their ability to produce IFN-*γ* in response to HLA-B4601-transfected COS-7 cells pulsed with a corresponding peptide or a negative control peptide, which can bind to HLA-B4601 molecules (p53_204–212_) (Azuma *et al*, 2003). The peptide-stimulated PBMCs were further incubated for more than 10 days, and their cytotoxicity against OSC20 (HLA-B46^+^) tumour cells, Kuma-1 (HLA-B46^−^) tumour cells, and PHA-blastoid T cells (HLA-B46^+^) was tested by a standard 6-h ^51^Cr-release assay, as reported previously (Azuma *et al*, 2003). To inhibit CTL activity, 20 *μ*g ml^−1^ of anti-HLA-class I (W6/32, IgG2a), anti-CD4 (Nu-Th/i, IgG1), anti-HLA-B, C (B1-23, IgG2a), anti-CD8 (Nu-Ts/c, IgG2a), and anti-HLA-class II (H-DR-1, IgG2a) mAbs were used (Azuma *et al*, 2003). Anti-CD14 (JML-H14, IgG2a) mAb served as a negative control. For the cold target inhibition assay, unlabeled cells of HLA-B46^+^ EBV-B cells were incubated with a corresponding or a control peptide for 2 h, washed, and added to the ^51^Cr-labelled targets at a cold : hot target ratio of 20 : 1. Two-tailed Student's *t*-test was employed for the statistical analysis for this entire study.

## RESULTS

### Identification of the gene and its expression

HLA-B46-restricted and tumour-reactive CTLs (OKB-CTLs) were used for cDNA screening to clone genes. Although the details regarding the characteristics of the OKB-CTLs were reported elsewhere (Azuma *et al*, 2003), we have tested the expression of CTL (CD3 and CD8) and NK cell markers (CD16 and NKD2D) on OKB-CTLs that were used as indicator cells for cloning of genes in this study, to exclude possible involvement of NK cell-mediated recognition of target cells. The surface phenotypes of the OKB-CTLs were CD3^+^ (>98%) CD8^+^(>90%) CD4^−^ (<5%) CD16^−^ (<1%) NKD2D^−^ (<1%). The results suggest no involvement of NK cell-mediated recognition of target cells in the case of the OKB-CTLs used in this study.

**Figure 1:**
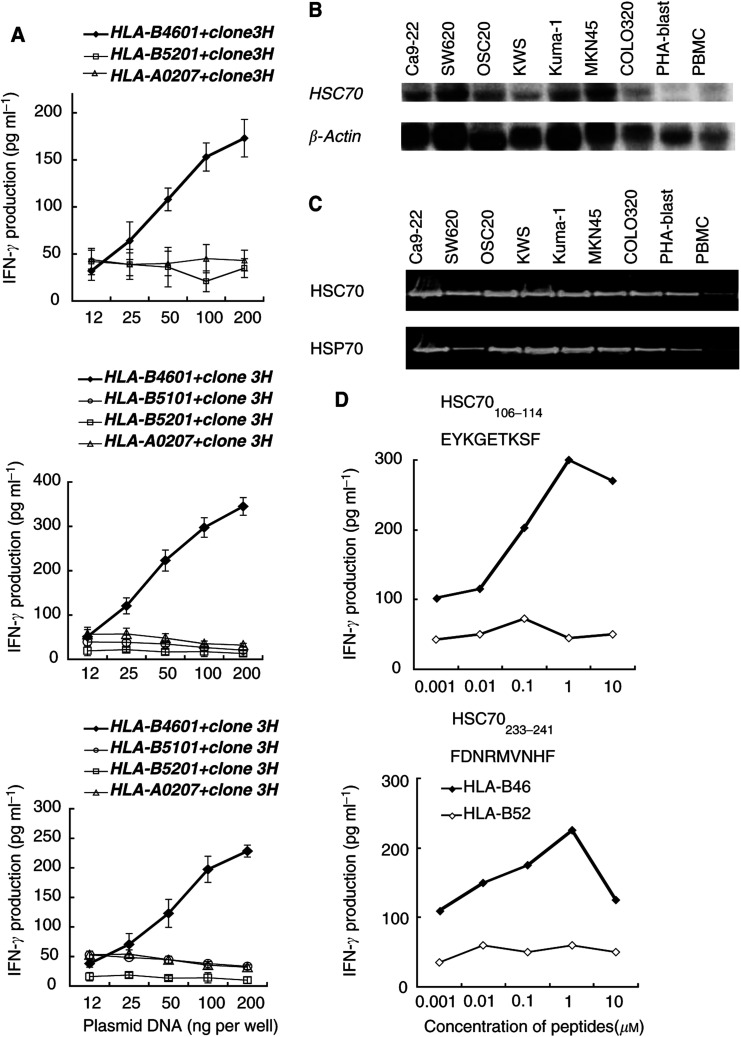
Determination of the gene. (**A**) Identification of a gene coding for tumour antigen. Amounts of 100 ng of a clone *3H* from SW620 cDNA libraries and 100 ng of the *HLA-B4601* gene were cotransfected into COS-7 cells. The *HLA-B5101* or *HLA-B5201* gene was used as a negative control. After incubation for 48 h, the cells were tested for their ability to stimulate IFN-*γ* production by the OKB-CTLs. COS-7 cells transfected with *HLA-A0207* and clone *3H* were also served as a negative control. The results of three separate experiments are shown in the figure. The background IFN-*γ* production in response to COS-7 cells (<100 pg ml^−1^) was subtracted. The values represent means of triplicate assays. ^*^P<0.05 by a two-tailed Student's *t*-test. (**B**) The mRNA expression of *HSC70.* The mRNA expression of HSC70 in various tumour cells and normal cells was analysed by Northern blotting. Representative results are given in the figure. (**C**) The expression levels of the constitutively expressed HSC70 or the stress-inducible HSP70 in the samples used for Northern blotting were investigated at the protein level by means of Western blotting with anti-Hsc70 or -Hsp70 mAb, respectively, to further investigate the expression of HSP family in tumour cells. (**D**) Recognition of an HSC70-derived peptide by the OKB-CTLs. Various doses of the peptides were loaded onto *HLA-B4601*-transfected COS-7 cells for 2 h followed by addition of the OKB-CTLs at an E/T ratio of 10 : 1. After 18 h incubation, the culture supernatants were collected in order to measure the IFN-*γ* production. The values represent of means of duplicate assays.

A total of 1 × 10^5^ cDNA clones from the cDNA library of SW620 tumour cells and *HLA-B4601* cDNA were cotransfected to the COS-7 cells followed by a test of their ability to stimulate IFN-*γ* production by the OKB-CTLs. After repeated experiments, one cDNA clone, *3H*, was identified. Representative results from the three different experiments are shown in Figure 1A

. The sequence of *3H* proved to be 2218 base pairs (bp), and it was almost identical to that of the stress-inducible HSPA8 (HSC70), although it was 36 bp shorter at the 5′-end and 6 bp shorter at the 3′-end than the registered sequence of HSPA8 (Accession No.: NM 006597). Neither mutation nor polymorphism was found in the sequence. Its open reading frame started from 43 to 1980 bp. It encodes 641 aa's and has 81% of homology at the aa levels with the stress-inducible heat shock protein 70 (HSA70) with 646 aa's.

The OKB-CTLs recognised COS-7 cells cotransfected with the clone *3H* gene and the *HLA-B4601* gene in a dose-dependent fashion. The results of three separate experiments are shown in Figure 1A. In contrast, the OKB-CTLs failed to recognise COS-7 cells cotransfected with the clone *3H* and the *HLA-B5201, -B5101*, or the *HLA-A0207* gene. The OKB-CTLs also failed to recognise COS-7 cells transfected with either the *HLA-B4601* gene or clone *3H* alone (data not shown). The mRNA expression of *clone 3H* in various tumour cells and normal cells was analysed by Northern blotting, and the representative result is shown in Figure 1B. It was highly expressed in Ca9-22, SW620, OSC20, Kuma-1, and MKN45 tumour cells. HLA-B46^+^ tumour cells were susceptible to lysis by the OKB-CTLs, as reported elsewhere (Azuma *et al*, 2003). In contrast, it was moderately expressed in KWS and COLO320, and weakly expressed in PHA-blastoid T cells and PBMCs. HLA-B46^+^ PHA-blastoid T cells were not lysed by the OKB-CTLs as reported previously (Azuma *et al*, 2003).

The expression levels of the constitutively expressed HSC70 or the stress-inducible HSP70 in the samples used for Northern blotting were investigated at the protein level by means of Western blotting with anti-Hsc70 or -Hsp70 mAb, respectively, to further investigate the expression of HSP family in tumour cells. HSP70 is a family of HSPs, and has 74 and 81% sequence homology with HSC70 at the nucleotide and aa levels, respectively. Expression levels of HSC70 in all the tumour cells and PHA-blast were higher than that in PBMCs, while those of HSP70 in all the tumour cells were higher than that in PHA-blast, which in turn was higher than that of PBMCs (Figure 1C).

### Identification of CTL-directed epitopes

**Figure 2:**
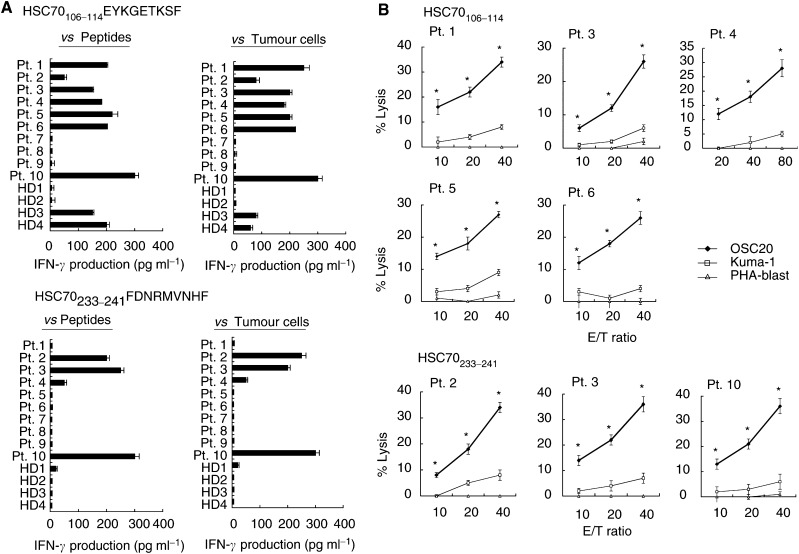
Determination of CTL epitope peptides. (**A**) Two peptides, HSC70_106–114_ and HSC70_233–241_, were tested for their ability to induce HLA-B46-restricted and tumour-reactive CTLs from the PBMCs of HLA-B46^+^ 10 cancer patients and four HDs. The details of the method are described in the Materials and Methods section. The data showed amounts of IFN-*γ* produced by peptide-treated PBMC in response to HLA-B4601-transfected COS7 cells pulsed with the corresponding peptides (left column) and OSC20 (HLA-B46^+^) tumour cells (right column) at an E/T ratio of 20 : 1. The background IFN-*γ* release in response to HLA-B4601-transfected COS7 cells pulsed with a control peptide (p53_204–212_) (<100 pg ml^−1^) was subtracted. The background IFN-*γ* release in response to Kuma-1 (HLA-B46) tumour cells (<50 pg ml^−1^) was also subtracted from the figure. The values represent the means of duplicate assays. (**B**) The cytotoxicity of the peptide-treated PBMCs from seven cancer patients against OSC20 (HLA-B46^+^), Kuma-1 (HLA-B46^−^), and PHA-blastoid T cells was confirmed by a 6-h ^51^Cr-release assay. Values represent the means of triplicate assays at three different E/T ratios. ^*^P<0.05 by a two-tailed Student's *t*-test.

**Figure 3:**
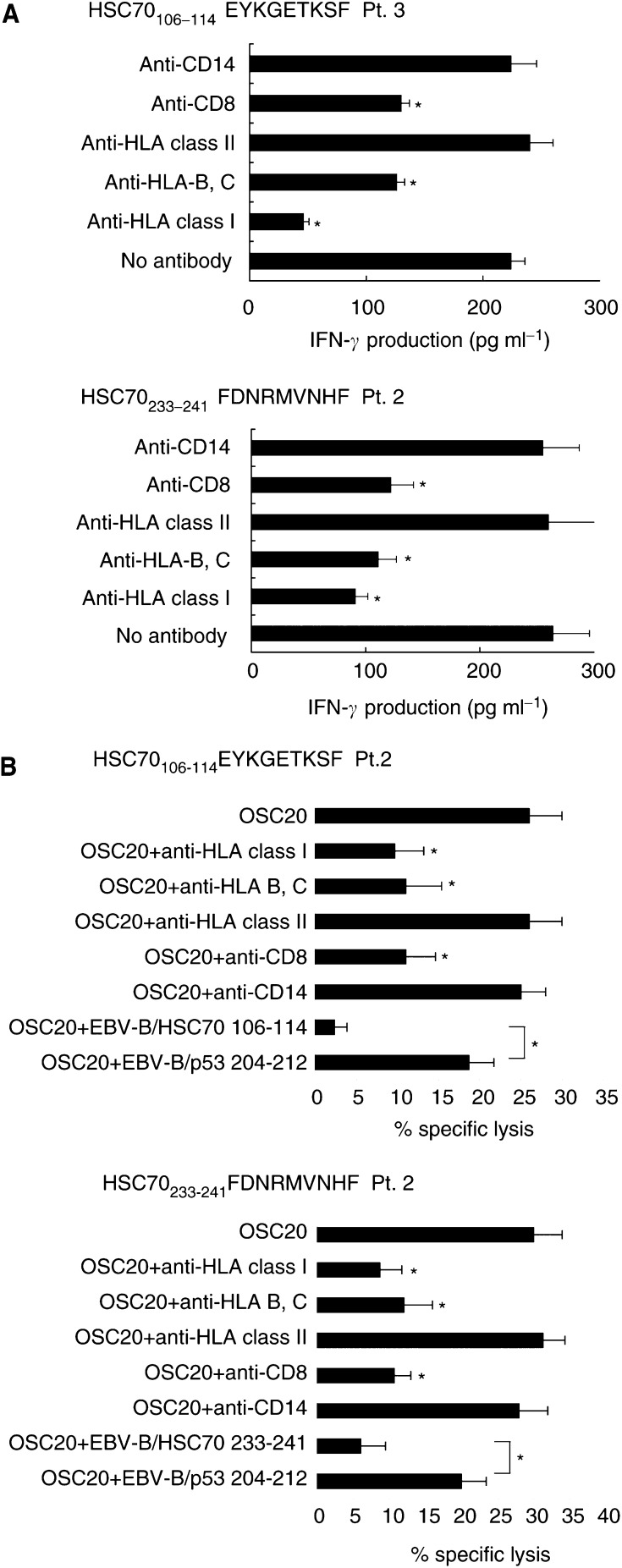
Inhibition tests and peptide specificity. (**A**) Inhibition test. IFN-*γ* production by the HSC70_106–114_ and HSC70_233–241_-stimulated PBMCs in response to OSC20 tumour cells was tested at an E/T ratio of 20 : 1 in the presence of 20 *μ*g ml^−1^ of anti-HLA class I, anti-HLA class II, anti-CD4, anti-CD8, anti-CD14, or anti-HLA-B, C mAb. Values represent the means of triplicate assays ^*^P<0.05 by a two-tailed Student's *t*-test. (**B**) Inhibition test and peptide specificity. Cytotoxicity by the peptide-stimulated PBMCs against OSC20 was tested at an E/T ratio of 20 : 1 in the presence of 20 *μ*g ml^−1^ of the mAb shown above. The CTL activity was inhibited by anti-CD8, anti-HLA class I, and anti-HLA-B, C mAb, but not by any other mAbs tested. For measurement of peptide specificity, an excess amount (20 *μ*g ml^−1^) of a corresponding peptide (HSC70_106–114_ or HSC70_233–241_) or a control peptide (p53_204–212_) was preloaded onto the ^51^Cr-labelled OSC20 tumour cells, which were used as target cells in a 6-h ^51^Cr-release assay. As cold cells, unlabelled EBV-B cells preloaded with either a corresponding peptide (HSC70_106–114_ or HSC70_233–241_), or a control peptide (p53_204–212_) were added to wells containing the ^51^Cr-labelled OSC20 tumour cells, at a cold-to-hot cell ratio of 20 : 1. Values represent the means of triplicate assays. ^*^P<0.05 by a two-tailed Student's *t*-test.

To identify the CTL-directed epitopes of HSC70, 10 kinds of HSC70-derived nonapeptides with HLA-B46-binding motifs were loaded on COS-7 cells transfected with *HLA-B4601* or *HLA-B5201* gene as a negative control, and then were tested for their ability to stimulate IFN-*γ* production by the OKB-CTLs. Two peptides, HSC70_106–114_ and HSC70_233–241_, were recognised by the OKB-CTLs in a dose-dependent fashion, and the highest IFN-*γ* production was observed at the concentration of 1 *μ*M peptide (Figure 1D). These two peptides were then tested for their ability to induce HLA-B46-restricted and tumour-reactive CTLs from the PBMCs of 10 HLA-B46^+^ cancer patients and 4 HD (Figure 2A

). Higher levels of IFN-*γ* production (>100 pg ml^−1^) by recognition of the corresponding peptide were observed in the following cases: HSC70_106–114_ peptide-stimulated PBMCs from six of 10 cancer patients and two of four HD; and HSC70_233–241_ peptide-stimulated PBMCs from three of 10 cancer patients and zero of four HD (Figure 2A). All of these PBMCs from cancer patients, but not from those of any HDs, also produced higher levels of IFN-*γ* production (>100 pg ml^−1^) in response to OSC20 cells (HLA-B46^+^HSC70^+^) but not in response to Kuma-1 cells (HLA-B46^−^HSC70^+^) (Figure 2A). The background IFN-*γ* production in response to the Kuma-1 tumour cells (<50 pg ml^−1^) was subtracted in the Figure 2A. Then, the cytotoxicity of the peptide-stimulated PBMCs from six cancer patients and those from two HDs was confirmed by a 6-h ^51^Cr-release assay, and the representative results of cancer patients were shown in Figure 2B. The PBMCs stimulated by the HSC70_106–114_ or HSP70_233–241_ peptides showed higher levels of cytotoxicity against the OSC20 cells, no or low levels of cytotoxicity against Kuma-1 cells, and no cytotoxicity against PHA-blastoid T cells (HLA-B46^+^) (Figure 2B). The peptide-stimulated PBMCs from a few cases (#1–#3) showed the low levels of cytotoxicity (5–10%) against Kuma-1 tumour cells at the higher effector to target (E/T) ratio, but it would be largely due to the cytotoxicity mediated by lymphokine-activated killer cells. In contrast, the HLA-restricted cytotoxicity against tumour cells was not obtained from the HSC70_106–114_ peptide-stimulated PBMCs from HD3 and HD4. Collectively, PBMCs from the majority (six of 10 cases) of cancer patients but none from HDs tested showed HLA-B46-restricted cytotoxicity against tumour cells after *in vitro* stimulation by at least one of these two peptides. Peripheral blood mononuclear cells activated with IL-2 alone without peptide stimulation from these patients did not show such cytotoxicity (data not shown). Cytotoxic T lymphocytes activity of the PBMCs stimulated with the HSC70_106–114_ and HSC70_233–241_ peptides was inhibited by anti-CD8, anti-HLA-B, C, and anti-HLA class I mAb but not by any other mAbs tested. Representative results of two cases are given in Figure 3A

. Their cytotoxicity against OSC20 tumour cells was also inhibited by anti-CD8, anti-HLA class I, or anti-HLA-B, C mAb, but not by any other mAbs tested. Representative results of Pt. 2 were shown in Figure 3B. Peptide specificity of CTL activity of the HSC70 peptide-stimulated PBMCs against HLA-B46^+^ tumour cells was confirmed by means of competition assay. Namely, the CTL activity against OSC20 cells was neutralised by the addition of unlabelled EBV-B cells preloaded with the correspondent peptide (HSC70_106–114_ in the upper right and HSC70_233–241_ peptide in the lower right column), but not by those preloaded with a control peptide (p53-derived peptide with HLA-B46-binding motif). Representative results are shown in Figure 3B.

## DISCUSSION

HSC70 is a highly conserved protein through various species; it is a member of the family of molecular chaperones and plays an important role in protein synthesis and folding, due to its ability to prevent misfolding and aggregation and to promote folding and translocation (Srivastava *et al*, 1998; Chen and Androlewicz, 2001; Udono *et al*, 2001; Wang *et al*, 2002). Antigenic peptides are usually generated by proteasomes and are translocated from the cytoplasm to the endoplasmic reticulum by the transporter associated with antigen processing. The chaperoned peptides can be channelled into the endogenous class I presentation pathway of a subset of professional antigen-presenting cells, which are then able to prime CD8-positive CTLs. Further, purified preparations of HSC70, gp96, HSP70, and calreticulin isolated from cancer cells have been shown to elicit cancer-specific immunity against a wide range of cancers in animal models (Tamura *et al*, 1997; Srivastava *et al*, 1998; Dressel *et al*, 1999; Robert *et al*, 2001; Udono *et al*, 2001). In this study, we have reported for the first time that *HSC70* gene encodes two nonmutated epitopes, at positions 106–114 and 233–241, capable of inducing HLA-B4601-restricted and tumour-reactive CTLs from PBMCs of the six of 10 epithelial cancer patients tested. These findings suggest the two ways of HSC70 involvement in the immune response against tumour: the binding and ‘carrying’ of antigenic peptides on the one hand and the antigenic capacities of HSC70 by themselves on the other hand. These results may facilitate the better understanding of molecular mechanisms involved in tumour-derived HSC-70-mediated induction of specific immunity and CTL activation. However, it is not clear yet whether HSC70 encodes antigenic peptides recognised by HLA-A class I molecules since HLA-B4601 molecules are exclusively expressed in Asians. It is also needed to investigate whether the other chaperones such as HSP70 encode HLA-B46-restricted CTL epitopes since three and one aa sequence of HSC70_106–114_ and HSC70_233–241_ peptides differ from the corresponding sequence of the HSP70, respectively. The other question is the breaking of immunological tolerance against the autologous HSC peptides. This question could partly be explained by the overexpression of the HSC70 in tumour tissues shown in this study. We reported that nonmutated self-antigens involved in cellular proliferation and thus are highly expressed in tumour cells such as SART1 and SART3 antigens often encode CTL epitope peptides (Shichijo *et al*, 1998, Yang *et al*, 1999, Ito *et al*, 2001). Although detailed mechanisms involved in these issues are not clear at the present time, our results, along with those of previous studies, suggest that the two HSC70-derived peptides are appropriate molecules in use for cancer vaccines to HLA-B46^+^ epithelial cancer patients.

Overexpression of the stress-inducible HSP70 has been shown in various types of cancers (Ciocca *et al*, 1993; Kaur and Ralhan, 1995; Ralhan and Kaur, 1995; Ciocca *et al*, 1998; Jolly and Morimoto, 2000; Zylicz *et al*, 2001) and are associated with poor prognosis of cancer patients. We showed in this study by both Northern and Western blot analyses that, not only the HSP70 but also the constitutively expressed HSC70 was highly expressed in epithelial cancer cell lines as compared to that of PBMCs. In addition, the HSC70 had the ability to induce CTL responses through binding and carrying antigenic peptides. The critical region of HSC70 for binding and carrying antigenic peptides was mapped to residues 280–385 in the ATPase domain (Udono *et al*, 2001), while the CTL epitopes were located at positions 106–114 and 233–241. Taken together, both the HSC70 and its peptides might be an appropriate candidate in use for specific immunotherapy for cancer patients.

The HLA-B46 allele is expressed exclusively in Asians, but not in other ethnic groups. This allele is expressed in 30% of Singapore Chinese, 28% of the Thai population, 9% of Japanese, and 8% of Koreans, whereas it is expressed in <1% of Caucasians, Blacks, and Indians (Imanishi *et al*, 1992, Barber *et al*, 1996). We have tested HLA-B expression at the serological level from 80 epithelial cancer patients, including 10 colon cancer patients, but because of its lower frequency in Japanese, PBMCs from only 10 HLA-B46^+^ cancer patients (five lung cancer and five prostate cancer patients) and four HLA-B46^+^ HD served as subjects for the CTL induction assay. Owing to the limitation of available PBMCs for the study, the experiments using these PBMCs were repeated twice in the patients' PBMCs and three times in the PBMCs from HDs. Although the amounts of IFN-*γ* produced by the peptide-stimulated PBMCs were relatively low, this is in part due to the small numbers of PBMCs used for the induction assay (10^5^ cells per well). Regardless of this limitation, however, all the data shown in this study were consistent and reproducible. In particular, the HLA-restricted cytotoxicity against tumour cells by means of the ^51^Cr-release assay was always obtained from the peptide-reactive PBMCs from six cancer patients by means of IFN-*γ*-release assay. In contrast, the HLA-restricted cytotoxicity against tumour cells was never obtained from the peptide-reactive PBMCs from two HDs by means of IFN-*γ*-release assay. Subsequently, CTL precursors reacting to HSC70 peptides and tumour cells might mainly be found in the circulation of substantial numbers of cancer patients, while the CTLs to autologous HSC70 peptides are under immunological tolerance in HDs. However, because of a limited numbers of samples, further studies on HLA-B46^+^ PBMCs from colon cancer or the other types of cancers as well as PBMCs from HDs are needed to confirm the results shown in this study.
